# Long-Term Coronary Outcomes and Follow-Up After Kawasaki Disease: Insights from a 25-Year Follow-Up Cohort

**DOI:** 10.3390/jcm15166155

**Published:** 2026-08-07

**Authors:** Antonio Musolino, Alessandra Marchesi, Giovanni Antonelli, Livia Gargiullo, Flavio Storelli, Giovanni Orso, Benedetta Benelli, Marta Ventura, Ludovica Ariaudo, Giulia Cafiero, Giulio Calcagni, Benedetta Leonardi, Michele Lioncino, Aurelio Secinaro, Riccardo Babini, Alberto Villani

**Affiliations:** 1 General Pediatrics, Infectious Diseases and Level II Pediatric Emergency Department Unit, IRCCS Bambino Gesù Children’s Hospital, 00165 Rome, Italy; antonio.musolino@opbg.net (A.M.); alessandra.marchesi@opbg.net (A.M.); livia.gargiullo@opbg.net (L.G.);; 2 Clinical Cardiology Unit, Division of Cardiac Surgery, IRCCS Bambino Gesù Children’s Hospital, 00165 Rome, Italy; giulia.cafiero@opbg.net (G.C.); michele.lioncino@opbg.net (M.L.); 3 Meyer Children’s Hospital IRCCS, 50139 Florence, Italy; flaviostorelli@gmail.com; 4 Pediatric Emergency Department, IRCCS Bambino Gesù Children’s Hospital, 00165 Rome, Italy; 5 School of Pediatrics, Tor Vergata University of Rome, 00133 Roma, Italy; benedetta.benelli@opbg.net (B.B.); marta.ventura@opbg.net (M.V.); 6 Department of Sciences of Public Health and Pediatrics, University of Turin, 10124 Torino, Italy; 7 Advanced Cardiothoracic Imaging Unit, IRCCS Bambino Gesù Children’s Hospital, 00165 Rome, Italy; 8 Independent Data Analyst and Software Engineer, Via Armando Diaz 10, 20881 Bernareggio, Italy

**Keywords:** Kawasaki disease, coronary artery aneurysms, pediatric cardiology, long-term follow-up, coronary Z-score, cardiac imaging, exercise stress testing, cardiac tomography

## Abstract

**Introduction:** Kawasaki disease (KD) is an acute systemic vasculitis and the leading cause of acquired pediatric heart disease in high-income countries. Coronary artery aneurysms (CAA) represent the most severe complication and drive long-term cardiovascular risk. Despite improved outcomes with early intravenous immunoglobulin therapy, follow-up strategies remain heterogeneous, particularly for patients showing CAA regression. Dynamic risk stratification based on coronary Z-scores has been proposed, but long-term real-world data are still needed to optimize surveillance. **Methods:** We conducted a single-center, retrospective study including pediatric patients (age 1 month–18 years) with KD complicated by CAA, followed at Bambino Gesù Children’s Hospital (Rome) between 1999 and 2024. Coronary involvement was assessed using Boston Z-scores of the right coronary artery, left main coronary artery, and left anterior descending artery. CAA severity over time was analyzed using a composite MAX SCORE (highest Z-score among coronary branches) along with the 1-YEAR MAX SCORE (highest MAX SCORE reached within the 1 year of disease). The distribution and timing of cardiac computed tomography angiography (CCTA) and exercise stress testing (EST) during follow-up were analyzed in relation to coronary severity. **Results:** Among 502 KD patients, 122 (24.3%) developed CAA; 113 were included in the analysis. Mean age at diagnosis was 24.6 months (M/F 3.5:1). Multivessel involvement was observed in 72%, most frequently affecting the left anterior descending artery. Long-term follow-up ≥10 years was available for 31.9% of patients. Most changes in coronary severity occurred within the first year after disease onset, with complete CAA regression in 76.1% of patients. Conversely, 53% of patients affected by giant aneurysms at 12 months showed persistent severe disease at last follow-up. EST (164 tests in 40 patients) was almost universally negative for inducible ischemia (163/164), whereas CCTA (47 exams in 35 patients) was preferentially performed early and in higher-risk patients. Test prescription correlated more closely with 1-YEAR MAX SCORE than with contemporaneous severity. Echocardiography showed systematic differences compared with CCTA for right coronary and left anterior descending artery Z-scores. **Discussion:** In our experience, early coronary status was closely associated with the intensity of long-term surveillance strategies in KD. The 1-YEAR MAX SCORE was associated with subsequent patterns of coronary evolution, and the continuous 1-year Maximum Z-score showed good discriminatory ability for persistent CAA on ROC analysis, pending external validation. While the low rate of positive findings on EST raises questions about its diagnostic yield in real-world practice, CCTA provided detailed anatomical characterization. Overall, these findings suggest that early coronary severity may help inform individualized, severity-driven follow-up strategies, warranting confirmation in prospective multicenter studies.

## 1. Introduction

Kawasaki disease (KD) is an acute systemic vasculitis predominantly affecting medium-sized arteries. Coronary artery aneurysms (CAA) represent the most severe complication, for which KD is the leading cause of acquired heart disease in pediatric patients in high-income countries [1,2]. Presence, severity, and persistence of CAA largely determine long-term cardiovascular risk, including thrombosis, arterial stenosis, myocardial ischemia and acute heart failure [3,4]. Over the past decades, advances in early diagnosis and in both 1- and second-line therapeutic schemes, along with standardized echocardiographic assessment, have significantly improved the outcomes [5,6,7].

Current international guidelines recommend risk stratification based on maximal CA Z-score to guide both chronic therapy and follow-up strategies: the American Heart Association (AHA) and Japanese Circulation Society (JCS) propose surveillance schedules based on CAA severity, incorporating serial imaging of CA and functional tests for inducible myocardial ischemia in selected patients [8,9].

Nevertheless, marked heterogeneity persists in real-world follow-up practices, especially in patients with partial or complete regression of CAA. While the presence of persistent or giant aneurysms clearly implies intensive long-term surveillance, the optimal duration, frequency and modality of follow-up remain a subject of debate for those patients showing resolution of CAA [10,11].

Furthermore, growing evidence suggests persistent endothelial dysfunction even after normalization of coronary diameters, but the clinical relevance of these vascular alterations remains uncertain and long-term observational studies have reported very low incidences of major cardiovascular events in patients with transient or regressed CAA; this justifies concerns about potential overuse of diagnostic testing, cumulative radiation exposure, healthcare costs and psychological burden for patients and families [12,13,14].

The choice of cardiac imaging modalities plays a central role in both the acute phase of KD and its follow-up. Transthoracic echocardiography remains the 1-line imaging modality for coronary assessment, while cardiac computed tomography angiography (CCTA or cardiac CT) offers superior visualization of distal coronary segments and detection of stenosis or thrombosis at the expense of radiation exposure and higher costs. Functional tests, including exercise stress testing (EST) or pharmacological stress imaging (echo—stress and adenosine stress CMR) are commonly used to screen for inducible ischemia, although their sensitivity in pediatric KD populations has been questioned [15].

Current guidelines allow flexibility in the selection of diagnostic tests, enabling clinicians to tailor investigations according to institutional expertise and availability of resources, thereby introducing a source of variability in clinical practice.

In this context, long-term real-world data describing follow-up strategies related to CAA evolution are still limited. The identification of simple, early predictors of long-term CA outcomes could help optimize surveillance protocols and promote a more individualized and resource-conscious approach.

The aim of this study was to analyze a large, single-center cohort of pediatric patients with KD complicated by CAA over an extended follow-up period (25 years), focusing on long-term coronary outcomes and on the longitudinal distribution of the main diagnostic tests performed for each patient.

## 2. Materials and Methods

### 2.1. Study Design and Population

We conducted a single-center, retrospective observational study including pediatric patients (aged 1 month to 18 years) diagnosed with Kawasaki disease (KD) complicated by coronary artery aneurysms (CAA), classified according to the American Heart Association (AHA) criteria for coronary artery involvement and Z-score-based risk stratification, and followed at Bambino Gesù Children’s Hospital (Rome, Italy) between 1 June 1999 and 1 June 2024 [8].

The diagnosis of KD was established according to the American Heart Association (AHA) criteria. All Kawasaki disease diagnoses were retrospectively reviewed and confirmed according to the American Heart Association (AHA) guidelines that were current at the time of the original diagnosis. Patients with incomplete clinical data or without documented CA involvement were excluded from the analysis.

According to the current AHA guidelines, a coronary artery aneurysm is strictly defined by a Z-score ≥ 2.5. However, because our cohort includes patients with a Z-score ≥ 2, the acronym “CAA” is used throughout this manuscript to indicate coronary artery abnormalities rather than aneurysms, consistent with many long-term Kawasaki disease studies.

Patient inclusion was based exclusively on anatomical coronary findings obtained by transthoracic echocardiography; data from other imaging exams or functional investigations, including exercise stress testing and stress imaging, were not used for diagnostic classification.

### 2.2. Data Collection

A dedicated database was created using Microsoft Excel^®^ to collect and process demographic, clinical, laboratory, and instrumental data.

Transthoracic echocardiography was the primary surveillance tool, routinely evaluating coronary dimensions (LMCA, LAD, and RCA Z-scores), ventricular function, and potential complications, whereas cardiac biomarkers were not systematically collected and were excluded from analysis. Advanced diagnostic investigations were performed selectively based on coronary severity, symptoms, and contemporary guideline evolution over the 25-year period.

Echocardiographic and advanced imaging reports were longitudinally reviewed for each patient throughout the available follow-up.

### 2.3. Coronary Assessment and Z-Score Normalization

Coronary artery involvement in Kawasaki disease is currently assessed using body surface area-adjusted Z-scores, which express the number of standard deviations by which a measured coronary diameter differs from the expected value in a healthy reference population. This approach allows standardized assessment of coronary artery abnormalities across pediatric patients of different ages and body sizes and is recommended by current international guidelines for the diagnosis and longitudinal monitoring of coronary involvement in Kawasaki disease [8,16].

According to the American Heart Association classification, coronary artery involvement is categorized as follows:

- Z-score < 2, normal coronary arteries;
- Z-score ≥ 2.0 to <2.5, coronary dilatation only;
- Z-score ≥ 2.5 to <5.0, small aneurysm;
- Z-score ≥ 5.0 to <10.0, medium aneurysm;
- Z-score ≥ 10.0 or absolute diameter ≥ 8 mm, giant aneurysm.

In our cohort, CAA were assessed using Z-scores of the main coronary branches—right coronary artery (RCA), left main coronary artery (LMCA) and left anterior descending artery (LAD)—measured by transthoracic echocardiography at disease onset and during follow-up [16]. Among the available normalization systems, the Boston Z-score model was selected. For old echocardiographic reports in which coronary diameters were expressed in millimeters, corresponding Z-scores were retrospectively calculated using the Boston Children’s Hospital online calculator (zscore.chboston.org; accessed on 12–13 April 2024).

Similarly, coronary diameters reported on cardiac computed tomography angiography (CCTA) were normalized to Boston Z-scores to allow direct comparison with corresponding echocardiographic measurements.

### 2.4. Definition of Coronary Severity Scores

As CAA could refer to one or more coronary artery branches, to facilitate longitudinal analyses, we converted the standard AHA coronary artery classification into an ordinal composite variable (MAX SCORE), representing the highest Z-score category observed among the three major coronary branches at each time point. The categories were derived directly from the AHA risk stratification system and were coded numerically to simplify longitudinal comparisons:

- MAX SCORE 0: Z-score < 2 (normal coronary arteries);
- MAX SCORE 1: Z-score between 2 and 2.5 (coronary dilatation only);
- MAX SCORE 2: Z-score between ≥2.5 and 5 (small aneurysm);
- MAX SCORE 3: Z-score between ≥5 and 10 (medium aneurysm);
- MAX SCORE 4: Z-score ≥ 10 (giant aneurysm).

This composite score was not intended to replace established clinical classifications but rather to provide a practical summary measure of overall coronary disease severity during follow-up. For the ROC analysis, the continuous 1-Year Maximum Z-score (rather than the corresponding ordinal MAX SCORE derivative) was used as the predictor variable, in order to preserve the full granularity of the underlying measurement and to allow identification of a data-driven optimal cut-off. The ordinal MAX SCORE classification was employed for all other descriptive analyses.

For statistical analysis, three additional indices were defined to assess CAA severity over time for each patient and its relationship with diagnostic testing (Table 1).

### 2.5. Follow-Up Time Points and Diagnostic Testing

Follow-up was structured according to predefined timepoints starting from disease onset: baseline echocardiogram at 0 weeks, 2 weeks, 4 weeks, 8 weeks, 4–6 months, 1 year, 1.5 years, 2 years, 3 years, and subsequently at regular intervals, with long-term follow-up available for up to ≥10 years for a subset of patients.

### 2.6. Statistical Analysis

Statistical analyses were performed using Python version 3.11 (Python Software Foundation, Wilmington, DE, USA), employing the SciPy (version 1.12.0) and Statsmodels (version 0.14.1) libraries. Continuous variables were expressed as median and interquartile range (IQR). Non-parametric statistical tests were used due to non-normal data distribution: we applied the two-tailed Mann–Whitney U test for comparisons between two independent groups and the Kruskal–Wallis H test for comparisons among more than two groups. When appropriate, post hoc pairwise comparisons were performed using Dunn’s test with Bonferroni correction for multiple testing. Statistical significance was set at *p* < 0.05.

Given the retrospective and exploratory nature of the study, the analyses were primarily intended to describe associations between coronary severity and follow-up practices rather than to establish formal predictive relationships. Consequently, the findings should be interpreted as exploratory and hypothesis-generating.

## 3. Results

### 3.1. Study Population and Follow-Up

A total of 122 patients were followed at our Centre for KD complicated by CA abnormalities during the retrospective observation period, which spanned 25 years (1999–2024).

Nine patients were excluded for lack of information or death during the acute phase of KD (one case), for a final number of 113 subjects included for the analysis.

**Figure 1:**
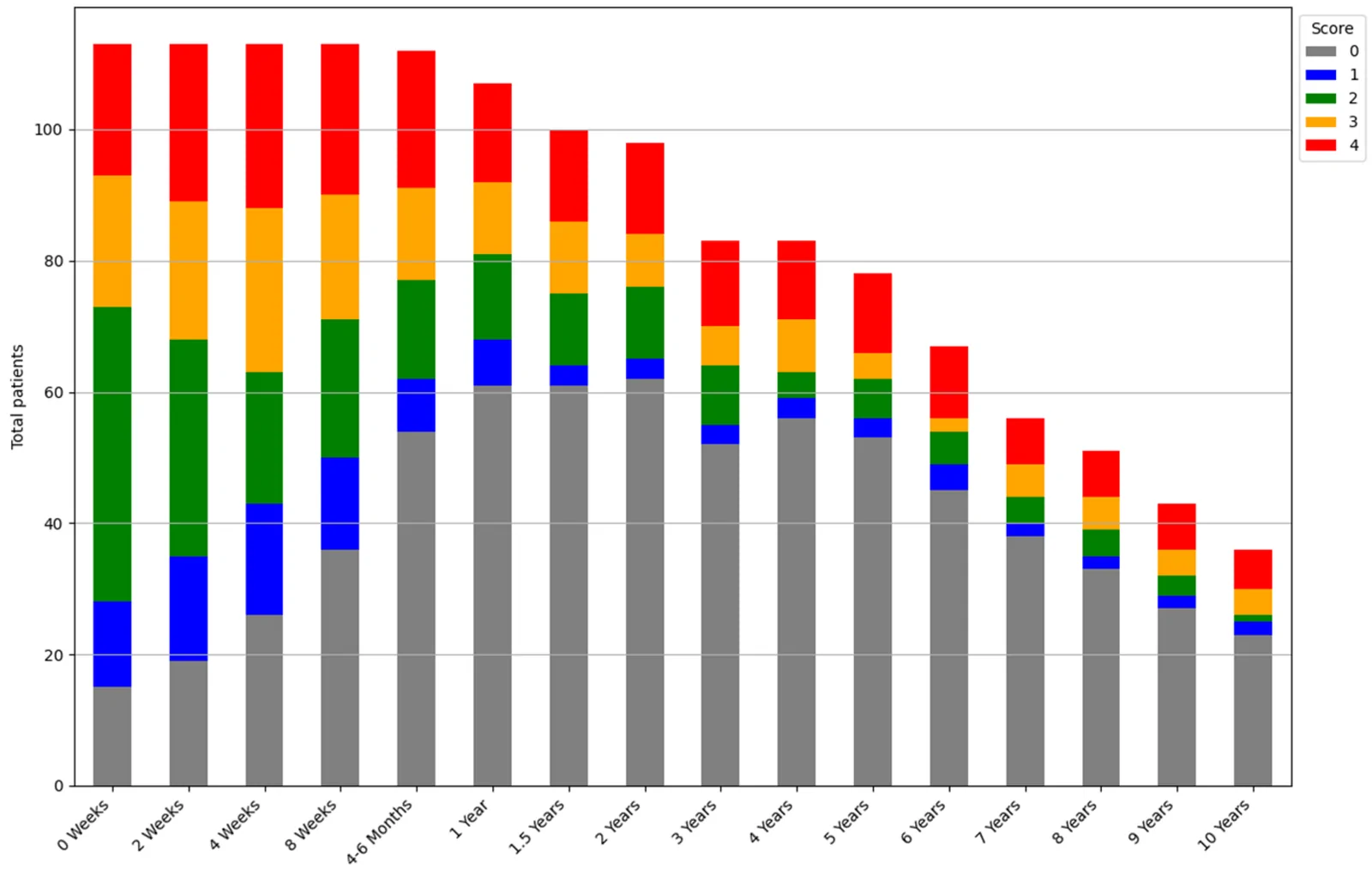
Patients’ distribution by MAX SCORE over available follow-up.

A complete follow-up duration of at least 10 years was available for 36 patients (31.9%), while follow-up data at 5 years were available for 79 patients (69.9%) (Figure 1).

### 3.2. Coronary Artery Involvement

Predominant involvement of the left CA was observed as expected, particularly affecting the left anterior descending artery (LAD) in terms of both frequency and severity of CAA. Giant aneurysms (MAX SCORE 4) were found in 31 patients (27.4%), while simultaneous involvement of at least two coronary branches was documented in 72% of patients, more than what is commonly reported in the literature.

### 3.3. Longitudinal Evolution of Z-Score During Follow-Up

Out of 113 patients, 86 (76.1%) showed a complete CAA regression by the end of their follow-up.

**Figure 2:**
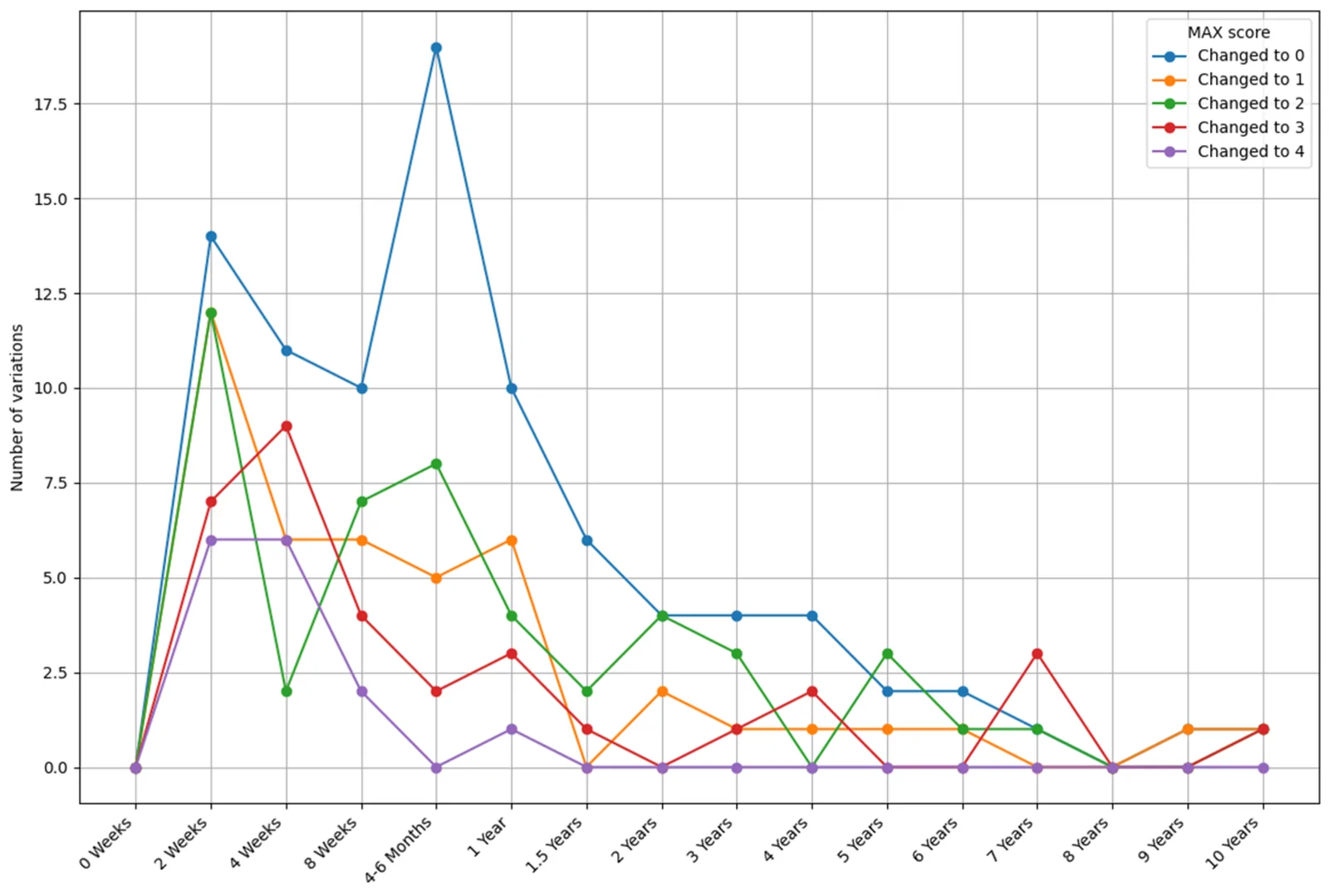
Number of MAX SCORE variations during follow-up.

Most changes in MAX SCORE class occurred within the 1 6 months after disease onset for the patients with lower CAA severity, whereas in those affected by giant aneurysms regression was more likely during the 1–12 months (Figure 2): overall, 54.8% (17/31) of patients with giant aneurysms displayed complete regression over the study period and it happened within the 1 year in all cases but one.

**Figure 3:**
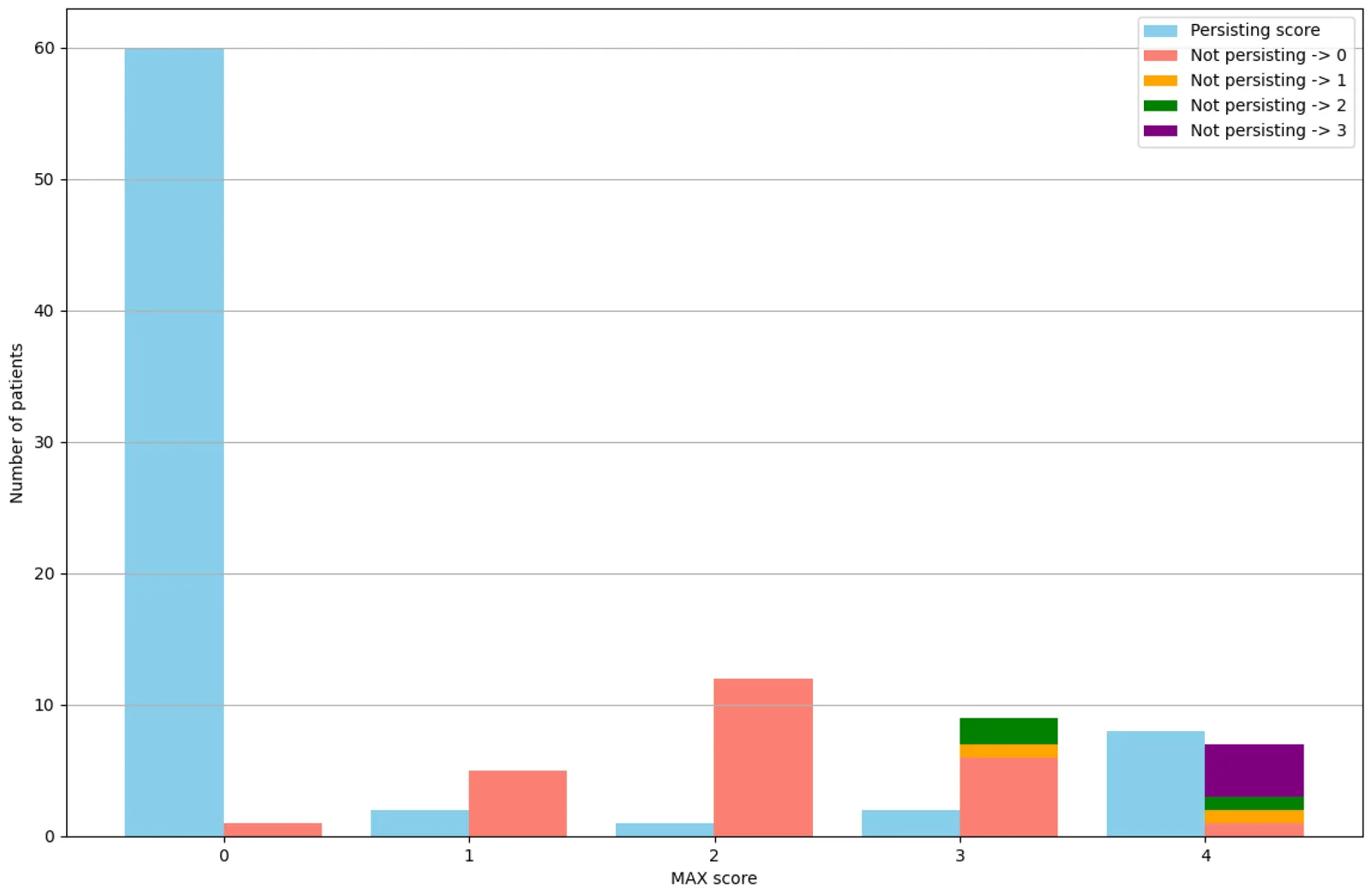
Longitudinal evolution of CAA in terms of persistence in the same MAX SCORE class after the first 12 months of disease.

After the 1 year of disease, nearly all patients affected by small and medium CAA (MAX SCORE 1, 2 or 3) showed subsequent complete regression of CAA (MAX SCORE 0); moreover, 71% of patients with Z-score < 2.5 at 12 months after disease onset, 92% of those with Z-score between 2.5 and 5 and 54.5% of those showing Z-score between 5 and 7.5 saw their CAA severity reducing over time, with a change in their MAX SCORE class. In contrast, after 12 months from disease onset, 15 patients in our cohort were showing giant aneurysms, for whom resolution of CAA was less likely: eight of them (53%) maintained succeeding severe coronary involvement over time, while the other group of patients (7/15) exhibited a progressive reduction in their MAX SCORE class, including one subject who experienced complete regression of CAA (Figure 3).

#### ROC Analysis for Prediction of Persistent CAA at 5 Years Follow-Up

**Figure 4:**
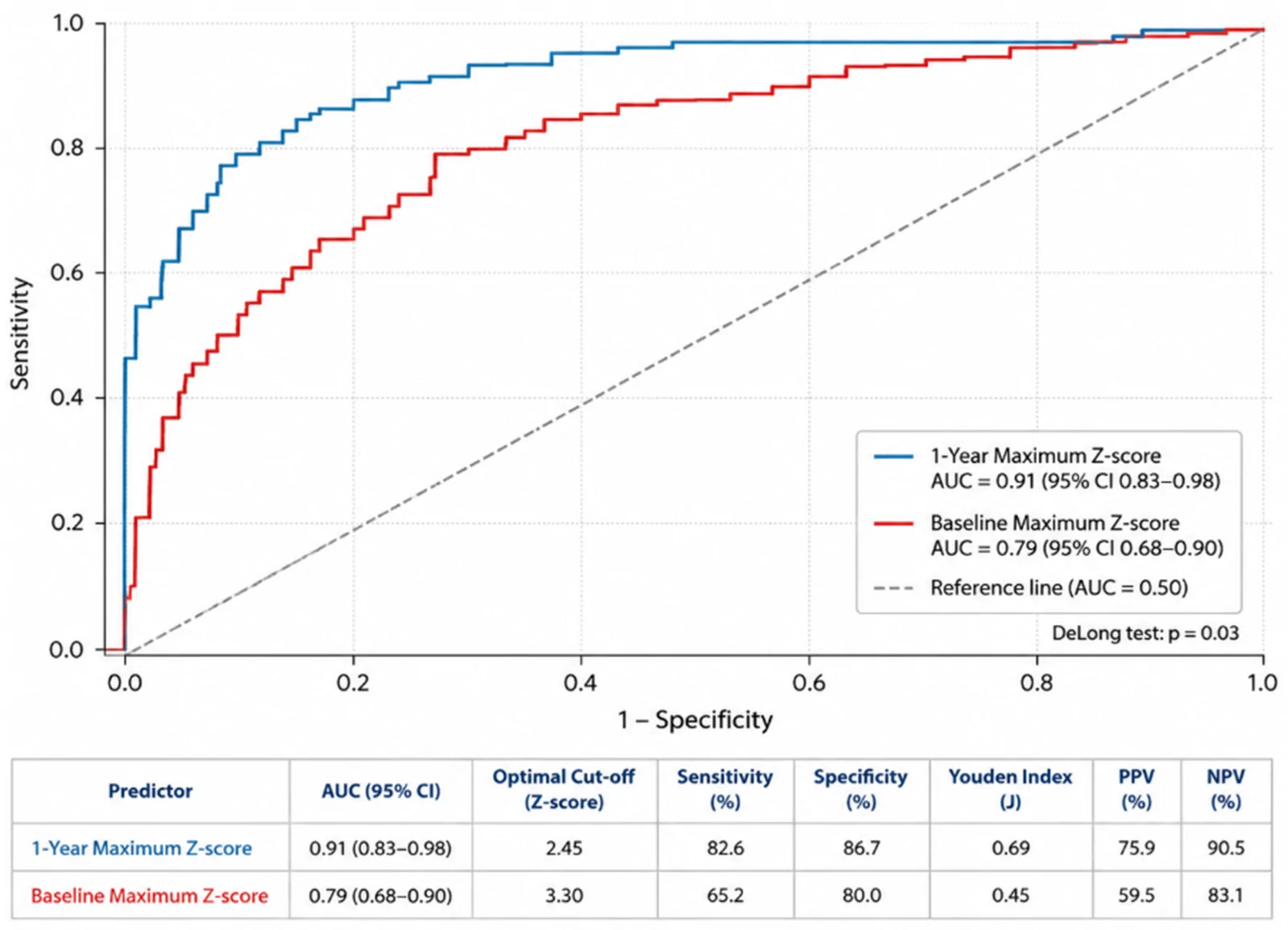
Receiver operating characteristic (ROC) curves for the 1-year maximum Z-score (blue) and the baseline (acute-phase) maximum Z-score (red) in predicting persistent coronary artery aneurysms at 5-year follow-up, in the subgroup of 79 patients with complete 5-year follow-up data (27 with persistent CAA, 52 with regression/no CAA). Circles indicate the Youden-optimal cut-off for each predictor. The 1-year maximum Z-score showed higher discriminatory performance than the baseline value (DeLong test, *p* = 0.03). AUC, area under the curve; CI, confidence interval; PPV, positive predictive value; NPV, negative predictive value.

To further explore the discriminatory ability of early coronary severity for long-term outcome, we performed a ROC analysis using the continuous 1-year maximum Z-score, from which the ordinal 1-YEAR MAX SCORE classification is derived. Receiver operating characteristic (ROC) analysis demonstrated that the continuous 1-year maximum Z-score discriminated patients with persistent coronary artery aneurysms at 5-year follow-up, with an AUC of 0.91 (95% CI 0.83–0.98). The optimal cut-off identified by the Youden index was a Z-score of 2.45, corresponding to a sensitivity of 82.6% and a specificity of 86.7% (PPV 75.9%, NPV 90.5%). The AUC of the 1-year maximum Z-score was significantly higher than that of the baseline (acute-phase) maximum Z-score (DeLong test, *p* = 0.03), suggesting that coronary status at 1 year provides greater discriminatory value for long-term outcome than the acute-phase measurement alone (Figure 4). This analysis was restricted to the subgroup of 79/113 patients (69.9%) with complete follow-up data available at 5 years, among whom 27 (34.2%) had persistent CAA at that point, whereas 52 (65.8%) showed regression of coronary involvement.

### 3.4. Longitudinal Distribution of Follow-Up Imaging Examinations

Exercise stress testing (EST) was performed in 40/113 patients (35.4%), accounting for a total of 164 examinations. All EST evaluations were negative for inducible ischemia or clinically relevant arrhythmias except for one case.

Coronary Computed Tomography Angiography (CCTA) was performed in 35/113 patients (31.0%), for a total of 47 examinations. Seventeen coronary stenoses were identified; however, the number of events was insufficient to perform meaningful statistical analyses regarding factors associated with stenosis development.

**Figure 5:**
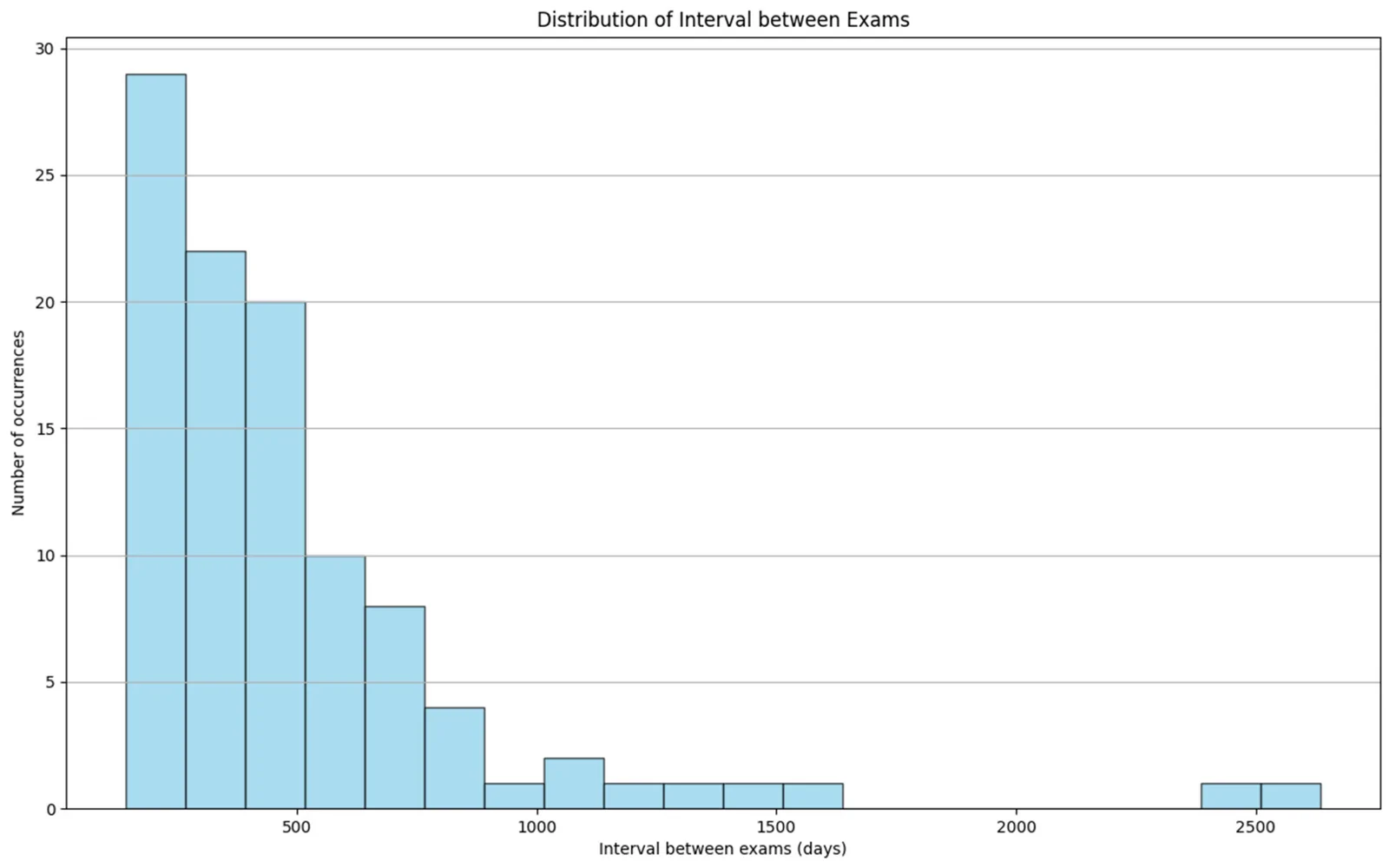
Timing of EST during follow-up according to the interval between tests (days).

**Figure 6:**
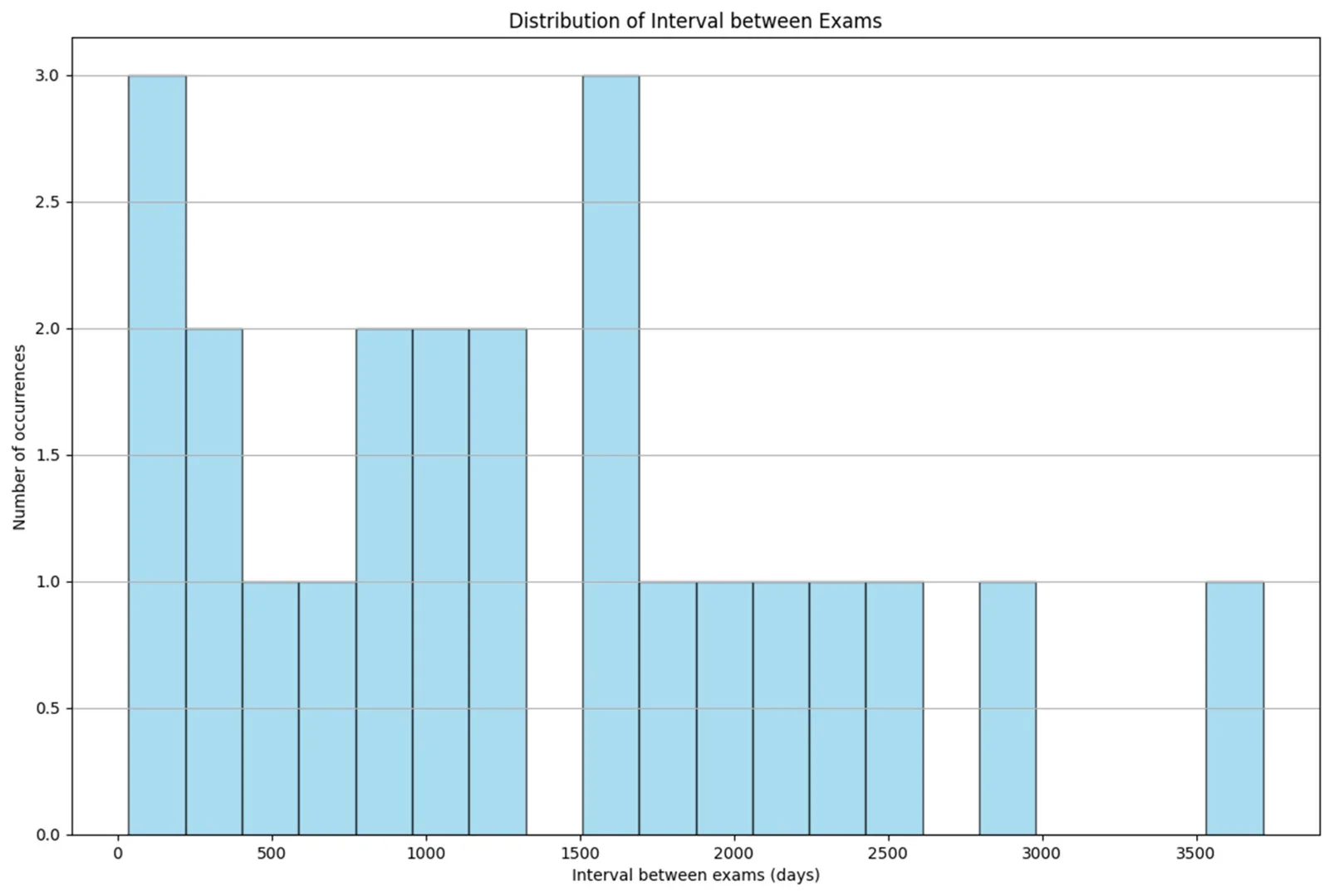
Timing of CCTA during follow-up according to the interval between scans (days).

Among patients undergoing repeated examinations, EST was generally performed at shorter intervals than CCTA. Because EST requires an adequate degree of patient cooperation, it was limited to children older than 6 years. In contrast, CCTA examinations were more evenly distributed throughout follow-up (Figure 5 and Figure 6).

### 3.5. Longitudinal Intervals of Exam Repetitions

**Figure 7:**
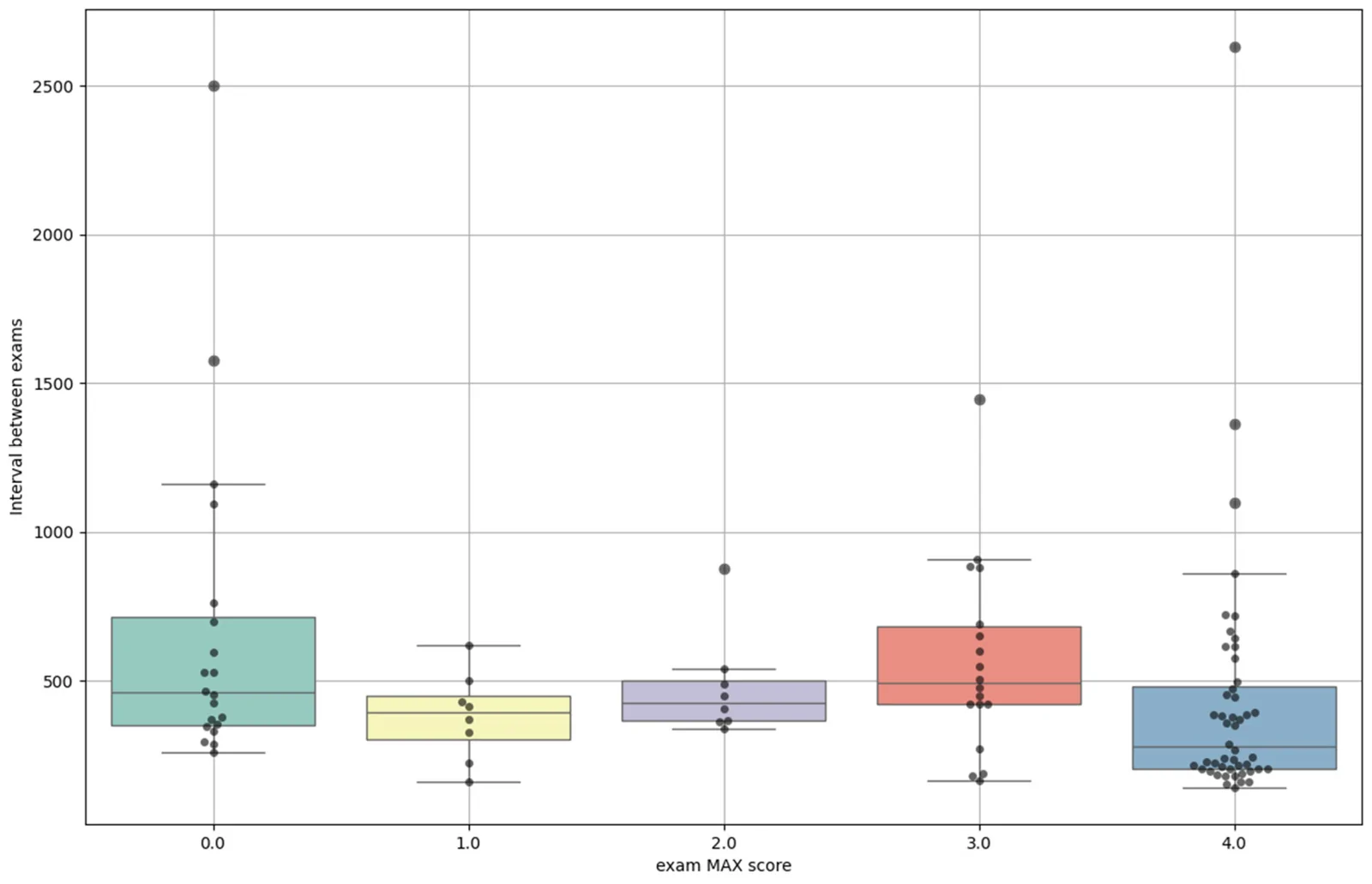
Timing of EST during follow-up according to exam MAX score (class).

Patients with giant aneurysms underwent EST at significantly shorter intervals than patients with lower coronary severity (Kruskal–Wallis H test, *p* = 0.002) (Figure 7).

**Figure 8:**
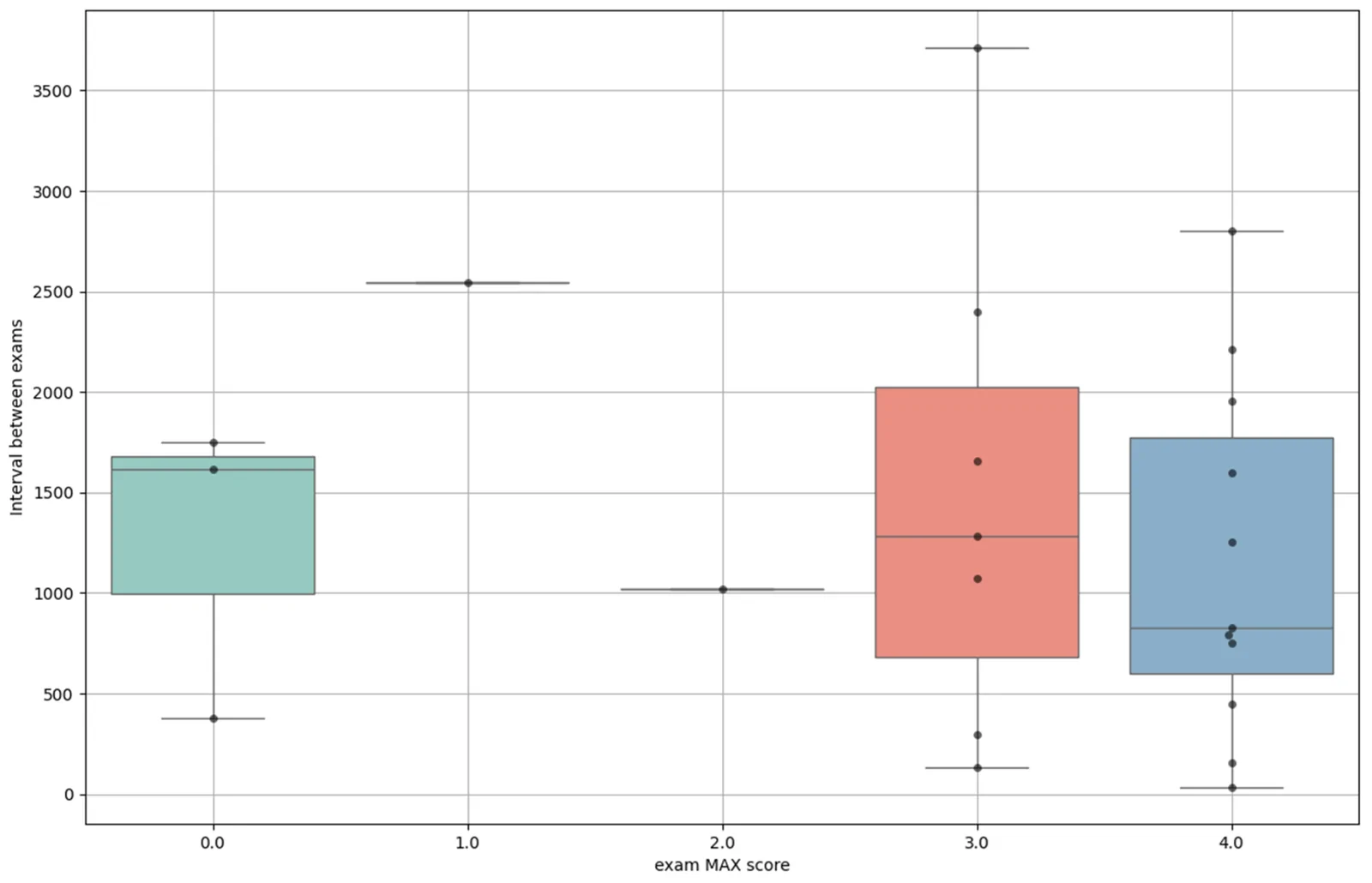
Timing of CCTA during follow-up according to exam MAX score (class).

No significant association was observed between coronary severity at the time of examination (exam-MAX SCORE category) and the interval between successive CCTA examinations (Kruskal–Wallis H test, *p* = 0.442) (Figure 8).

### 3.6. Concordance Between Echocardiography and Coronary CCTA at Disease Onset

**Figure 9:**
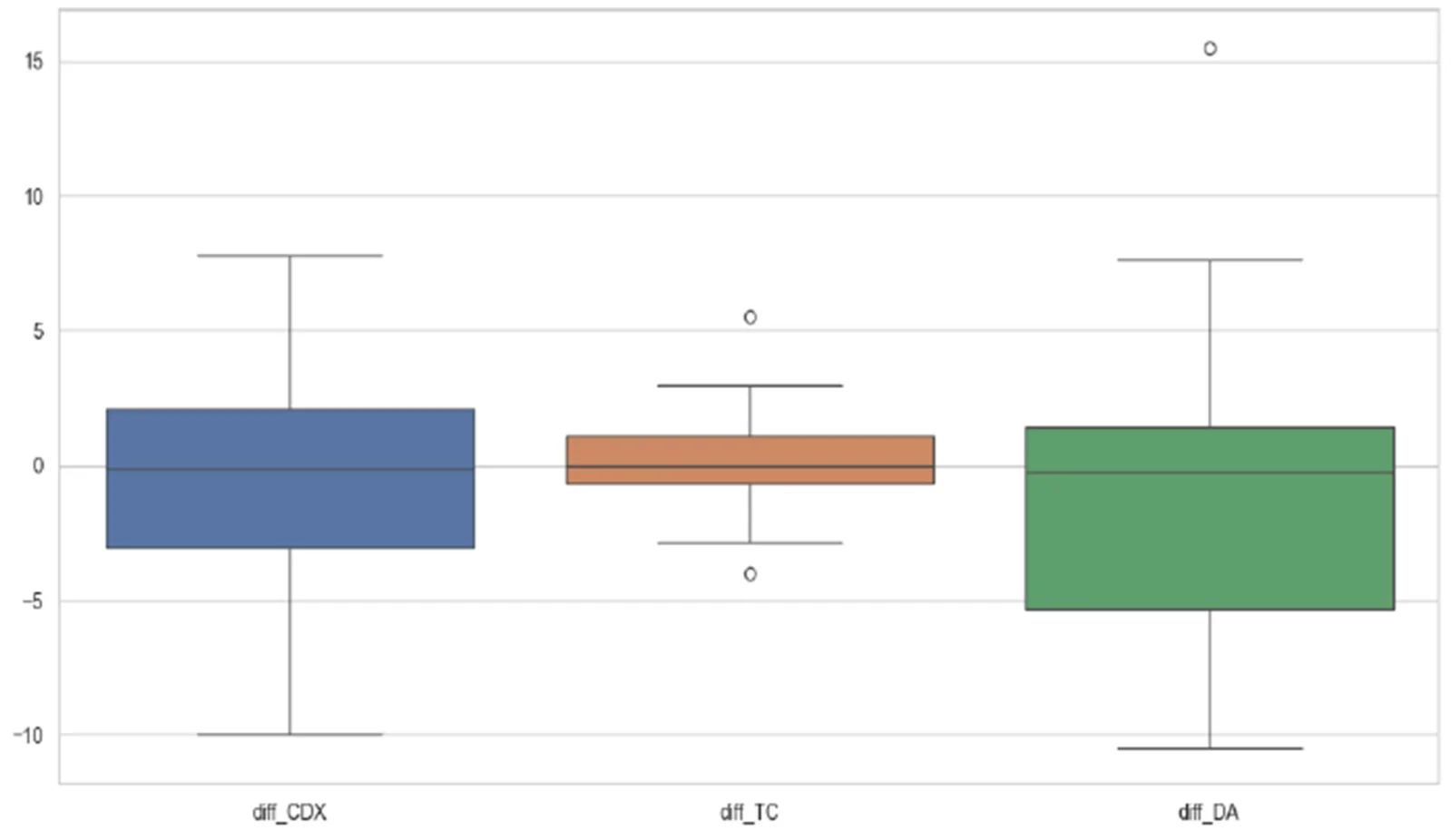
Boxplot for comparison of Z-score values obtained by CCTA and echocardiography for main coronary branches (right coronary artery-CDX; left main coronary artery—TC; left anterior descending artery—DA).

**Figure 10:**
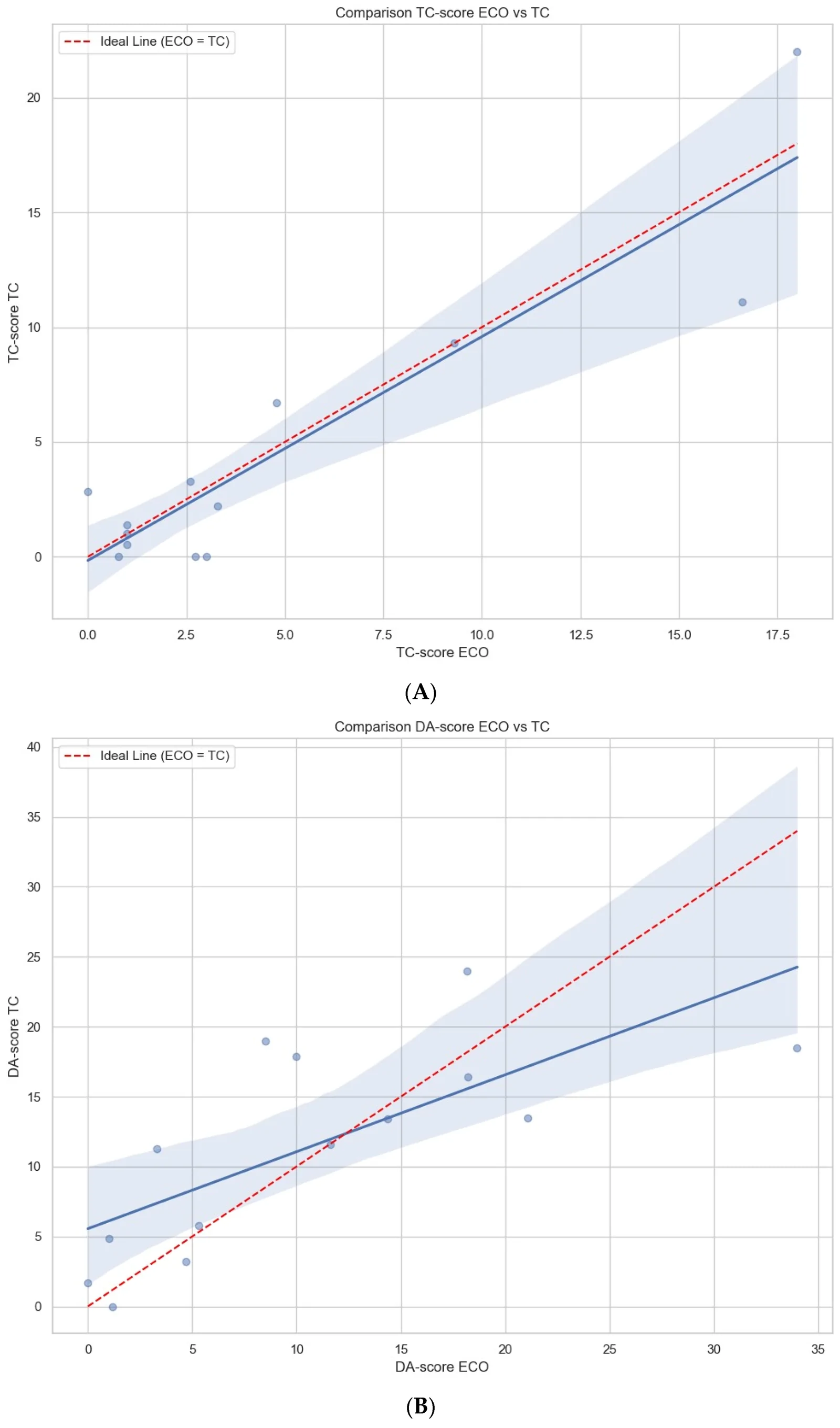

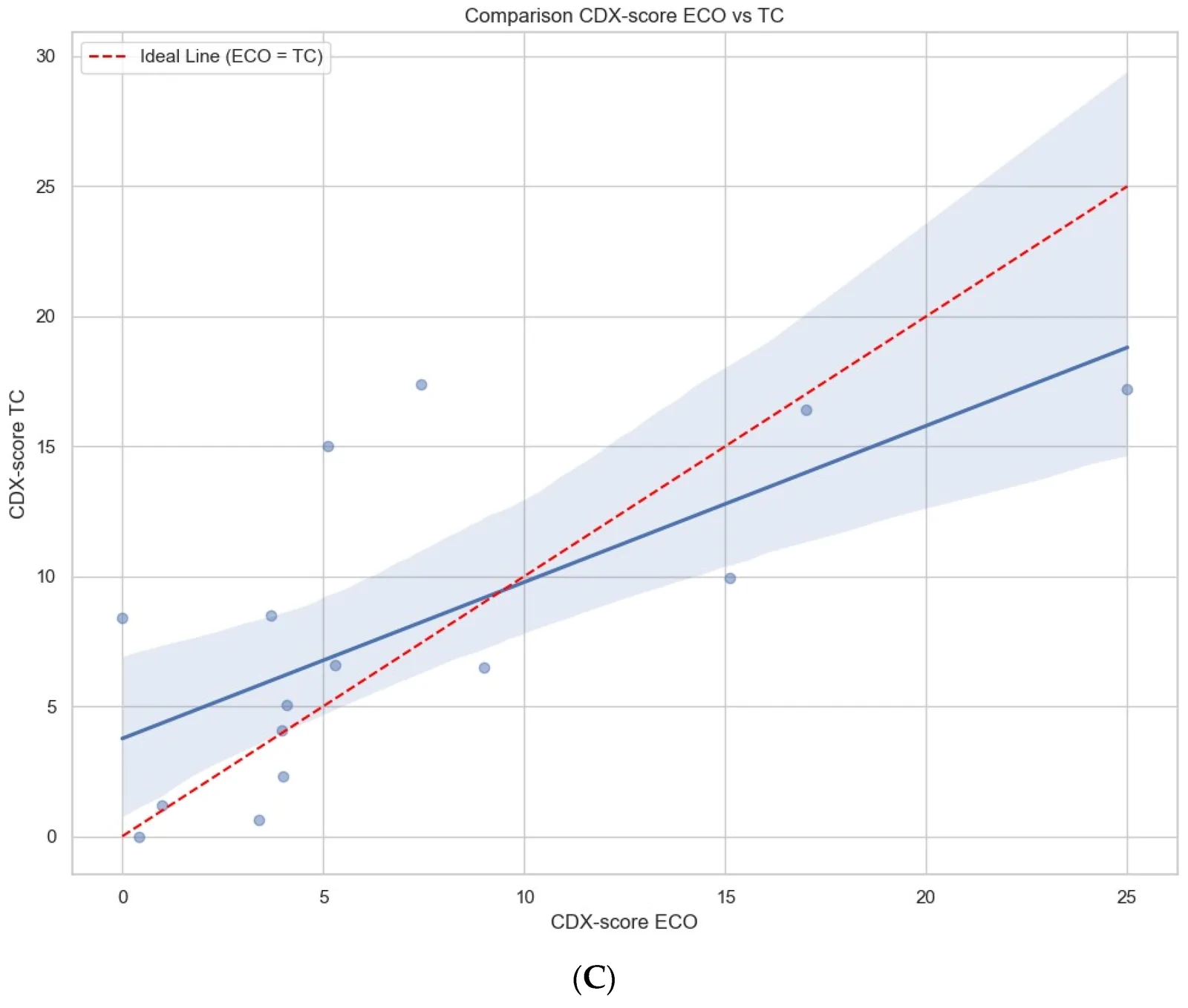
Linear regression analysis of Z-score values obtained by CCTA and echocardiography for LMCA (**A**), LAD (**B**), RCA (**C**). The blue line indicates the fitted linear regression line, and the shaded blue area represents the 95% confidence interval.

Comparison of Z-scores obtained by echocardiography and CCTA at disease onset revealed a closer relationship for LMCA measurements than for RCA and LAD values. These exploratory findings are illustrated in Figure 9 and Figure 10A–C.

### 3.7. Additional Second-Level Investigations

Additional second-level investigations were performed in selected patients and are reported descriptively. These included coronary angiography, cardiopulmonary exercise testing, stress SPECT, stress cardiac magnetic resonance imaging (stress CMR), and stress echocardiography, with limited case numbers for each modality (Table 2).

## 4. Discussion

Medium- and long-term follow-up of patients affected by KD complicated by CAA remains a debated issue at an international level, especially for those showing partial or complete regression of coronary involvement [17,18].

The most recent international guidelines provide risk-stratified recommendations while allowing flexibility in the choice and timing of diagnostic investigations, leading to heterogeneous real-world follow-up strategies across centers [19].

Our analysis, spanning a 25-year period and multiple guideline eras, provides a real-world perspective on long-term follow-up practices in pediatric patients with KD complicated by CAA. Our data highlight heterogeneity in surveillance strategies over the observation period, reflecting the absence of a universally standardized approach. Understanding these patterns is essential to contextualize clinical decision-making and may help develop more rational and risk-adapted follow-up protocols.

Longitudinal analysis of coronary Z-scores in our cohort of patients confirmed the known natural history of KD, with most changes in CAA severity occurring within the 1 year after disease onset: more specifically, complete CAA regression was mainly observed in patients with lower MAX SCORE values, whereas patients with severe aneurysms at one year showed a substantial risk of persistent coronary disease.

Early coronary involvement appeared to be closely associated with both long-term coronary evolution and follow-up strategies in our cohort [20].

In our cohort, the 1-YEAR MAX SCORE was associated with subsequent coronary evolution and with the intensity of long-term surveillance. Patients with persistent giant aneurysms at 12 months showed the highest probability of maintaining severe coronary involvement during follow-up.

ROC analysis further supported the discriminatory ability of the 1-year maximum Z-score for identifying patients at increased risk of persistent coronary artery aneurysms, and its performance appeared superior to that of the baseline acute-phase Z-score. Nevertheless, these findings derive from a retrospective single-center cohort and should be interpreted as hypothesis-generating; external, prospective validation is required before any diagnostic or predictive cut-off can be considered for clinical use.

From a practical perspective, the coronary status observed at one year may help support risk-adapted surveillance strategies. Patients showing complete regression of coronary abnormalities or only minimal residual coronary involvement at 12 months experienced very limited subsequent changes in coronary severity, suggesting that a gradual reduction in the intensity of follow-up investigations may be appropriate in selected low-risk patients, in accordance with current guideline recommendations. Conversely, patients with persistent giant aneurysms at one year represented the subgroup most likely to maintain significant coronary disease over time and therefore may benefit from continued intensive surveillance, including periodic advanced imaging and functional assessment.

Notably, the 1-YEAR MAX SCORE appeared to correlate more closely with the longitudinal distribution of follow-up investigations than contemporaneous coronary status (exam-MAX SCORE), suggesting that follow-up strategies in our cohort were largely influenced by early coronary severity, even among patients who subsequently experienced partial or complete regression of coronary abnormalities.

Notably, analyses based on the OVERALL MAX SCORE yielded findings largely overlapping with those obtained using the 1-YEAR MAX SCORE, likely because peak coronary Z-scores were reached within the 1 year of disease in most patients. Therefore, the 1-YEAR MAX SCORE may represent a surrogate marker of long-term coronary severity warranting further validation.

The latest AHA 2024 updates on Kawasaki disease build on this concept, recommending that long-term follow-up incorporate not only early risk stratification but also ongoing, dynamic assessment of coronary artery changes, introducing a temporal dimension that complements the real-world approaches used in our cohort.

Literature reports that patients without coronary complications or with regression of aneurysms have an extremely low incidence of cardiovascular events, although subclinical endothelial dysfunction such as calcification can persist [21,22]; this supports the possibility of tailoring follow-up intensity according to long-term coronary risk, in line with current guideline recommendations [23].

The high number of EST examinations observed in our cohort, despite the low prevalence of positive findings, reflects the real-world follow-up strategies adopted during the study period; notably, EST prescription did not appear to correlate closely with CAA severity. These observations are consistent with current guideline recommendations, which recognize the limited sensitivity of exercise electrocardiography in patients with Kawasaki disease. However, given the retrospective nature of the study, the absence of standardized indications for repeated testing and the evolution of guideline recommendations over the 25-year study period, these findings should be interpreted as descriptive of clinical practice but further studies are warranted to better define the optimal timing of EST within long-term surveillance strategies.

On the other hand, for what concerns structural imaging exams, CCTA was performed in more selected cases and played a greater role in patients with higher coronary Z-scores, at younger ages and prevalently during the early phases of follow-up. Longitudinally, CCTA examinations for a single patient tended to be spaced apart over time, reflecting a cautious approach aimed at balancing diagnostic yield and radiation exposure and costs. Indeed, the extensive use of diagnostic tests observed in our cohort highlights the complexity of long-term surveillance in patients with coronary involvement; in a broader context of healthcare sustainability, a more selective and appropriate use of examinations may help reallocate resources to other follow-up priorities [24].

Therefore, our findings may contribute to future discussions regarding risk-adapted surveillance strategies. Additionally, comparison of echocardiographic and CCTA-derived coronary measurements suggested greater variability for LAD and RCA than for LMCA [25,26]; these findings align with known limitations of echocardiography in assessing coronary measures (especially due to inter- and intra-observer variability) and could support a major use of advanced imaging techniques in patients with significant or complex coronary involvement, also considered the reduction in radiation exposure with new CCTA techniques [27].

In our cohort, other second-level investigations (including coronary angiography, single-photon emission computed tomography and CMR) were selectively performed in a few cases and were described without formal statistical analysis. More in detail, coronary angiography identified significant stenoses in nearly half of examined patients (6/13), confirming its role as the reference standard for detailed anatomical assessment in advanced disease [28]. CMR revealed ischemic findings without associated ventricular dysfunction in 4 out of 5 examined patients. In addition to the absence of radiation exposure, CMR offers important advantages including the assessment of ventricular function, myocardial perfusion, and myocardial tissue characterization. However, practical limitations such as prolonged acquisition times, the frequent need for sedation in younger children, limited availability, and lower spatial resolution for the assessment of distal coronary segments and advanced coronary stenosis compared with CCTA may restrict its widespread use in this population [29].

From a broader perspective, CMR should be regarded as a complementary rather than competing imaging modality in the long-term follow-up of Kawasaki disease. While CCTA remains particularly valuable for detailed anatomical assessment of coronary artery stenosis, calcification, thrombosis, and distal coronary segments, CMR provides com-prehensive functional evaluation and myocardial characterization. The optimal integration of CCTA and CMR within long-term surveillance strategies remains an area of active investigation and will likely evolve with ongoing technological advances. In addition, only a limited number of patients underwent CMR evaluation in our cohort, preventing any meaningful comparison between CMR and other imaging modalities. Consequently, our findings should not be interpreted as supporting one advanced imaging technique over another, but rather as reflecting real-world utilization patterns within a single-center experience.

This study presents some limitations, such as its single-center and retrospective nature; our findings may thus reflect local practices and not be fully generalizable to other settings with different organizational models and technical resources. Although the overall cohort size was adequate, the number of patients undergoing advanced imaging modalities was relatively small, limiting the possibility for detailed subgroup analyses. In addition, the low incidence of major adverse cardiac events restricted our ability to analyze them, and the observational design prevented us from establishing causal relationships between surveillance strategies and outcomes.

An additional limitation relates to the statistical approach used in the present study. Because patients contributed repeated echocardiographic assessments and, in some cases, repeated functional and imaging investigations throughout follow-up, the resulting data structure was inherently longitudinal. Although our analyses were intended primarily to provide a descriptive overview of real-world follow-up practices and coronary evolution, the statistical methods applied did not explicitly account for within-patient correlations over time. More sophisticated approaches, such as mixed-effects models, ordinal longitudinal analyses, or time-to-event methods, may provide a more robust evaluation of coronary artery evolution and should be considered in future studies. Furthermore, although the 1-YEAR MAX SCORE was associated with subsequent coronary evolution and with the intensity of follow-up investigations, its prognostic value should be considered exploratory and requires confirmation in prospective multicenter studies specifically designed for predictive validation. For ROC analysis, the optimal cut-off derived from the Youden index was identified within the same cohort used to estimate model discrimination, without internal validation (e.g., bootstrap resampling or cross-validation); therefore, this cut-off should be regarded as hypothesis-generating pending external validation in an independent cohort.

Finally, the comparison between echocardiographic and CCTA-derived Z-scores was based on descriptive and regression analyses rather than formal agreement methods such as Bland–Altman analysis or intraclass correlation coefficients. Therefore, no definitive conclusions regarding inter-modality agreement can be drawn from the present data.

Given the retrospective and exploratory nature of the study, our findings should primarily be interpreted as describing patterns of coronary evolution and follow-up practices rather than establishing formal prognostic, economic, or management recommendations.

## 5. Conclusions

In this real-world cohort of pediatric patients with Kawasaki disease complicated by coronary artery aneurysms, long-term follow-up practices appeared to be influenced primarily by the severity of coronary involvement during the first year after disease onset, often regardless of subsequent coronary regression. The 1-YEAR MAX SCORE was associated with subsequent patterns of coronary evolution and with the intensity of long-term surveillance in this cohort. The continuous 1-year maximum Z-score further showed good discriminatory ability for persistent coronary artery aneurysms on ROC analysis, which appeared superior to that of the baseline acute-phase value (AUC 0.91 vs. 0.79). However, these findings require confirmation in prospective, multicenter studies before any diagnostic threshold could be considered for routine clinical implementation.

While Coronary Computed Tomography Angiography was generally reserved for patients with more severe coronary involvement, exercise stress testing was frequently repeated despite a low prevalence of positive findings; further studies are warranted to better define its optimal role and timing within long-term surveillance strategies.

## Figures and Tables

**Table 1:** Derived indices of MAX SCORE for longitudinal analysis of coronary artery involvement in Kawasaki disease.

Index	Definition	Purpose
1-YEAR MAX SCORE	Highest MAX SCORE reached within the first 12 months after disease onset	Time-independent index, possible early predictor
Overall-MAX SCORE	Highest MAX SCORE observed throughout the entire follow-up for each patient	To evaluate the predictive reliability of 1-YEAR MAX SCORE
Exam-MAX SCORE	MAX SCORE recorded at the last echocardiographic examination preceding each EST or CCTA	To assess coronary severity at the time of diagnostic testing

**Table 2:** Additional diagnostic tests and results in the study cohort.

Modality	N Patients (%)	Findings
Coronary angiography	13 (10.6%)	Coronary stenosis > 70% in 6 (46.2%)
Cardiopulmonary exercise test (VO_2_/VCO_2_)	8 (6.5%)	Negative (100%)
Stress SPECT (dipyridamole)	8 (6.5%)	Ischemic damage in 3 (37.5%) with mild LV EF reduction in 2 of them
Stress CMR (adenosine)	5 (4.1%)	Myocardial ischemia in 4 (80%) without LV dysfunction
Stress echocardiography	2 (1.6%)	Negative (100%)

## Data Availability

The raw data supporting the conclusions of this article will be made available from the corresponding author upon reasonable request.
